# Dietary habits, oral impact on daily performance and type 2 diabetes: a matched case-control study from Sudan

**DOI:** 10.1186/s12955-017-0686-9

**Published:** 2017-05-22

**Authors:** Hasaan G. Mohamed, Kamal Mustafa, Salah O. Ibrahim, Anne N. Åstrøm

**Affiliations:** 1 Department of Oral Rehabilitation, Faculty of Dentistry, University of Khartoum, El-Qasr Street, 11123 Khartoum City, Sudan; 2 Department of Clinical Dentistry, Faculty of Medicine and Dentistry, University of Bergen, Bergen, Norway

**Keywords:** OHRQoL, Food-frequency questionnaire, Diabetes, Oral health

## Abstract

**Background:**

It is evident that social and behavioural factors influence on individuals’ general health and quality of life. Nevertheless, information about the influence of dietary habits on oral health-related quality of life is limited; especially among patients with type 2 diabetes (T2D). The aim of this study was to examine the influence of dietary habits and clinical oral health indicators on oral health-related quality of life in individuals with and without T2D.

**Methods:**

A total of 149 T2D cases and 298 controls were recruited for this age and gender matched case-control study. Questionnaire-guided interviews were conducted to collect data about socio-demographic characteristics, consumption of food items per week (milk, meat, eggs, vegetables, fruits, sweets and bread) and oral impact on daily performance (OIDP). Plaque index, bleeding on probing, probing depth, tooth mobility, decayed, missing and filled teeth index (DMFT) and root caries were recorded.

**Results:**

Difficulty with eating and sleeping were more frequently reported by T2D cases (23.5% and 16.1%, respectively) than by the controls (10.7% and 5.0%, respectively) (*P* < 0.01). After adjusting for diabetic status, plaque index, bleeding on probing, probing depth, tooth mobility, root caries, and missing teeth, those with high consumption of milk and sweets, were more likely than those with low consumption to report any oral impact (OIDP > 0). The corresponding ORs were 1.23 (1.01–4.89) and 2.10 (1.08–4.09), respectively. Participants with low consumption of meat and vegetables were more likely than their counterparts with high consumption to report any oral impact. The corresponding ORs were 0.46 (0.25–0.83) and 0.38 (0.17–0.87), respectively. There was a significant interaction between diabetic status and meat consumption as well as between diabetic status and bread consumption.

**Conclusions:**

Oral impacts were more frequently reported in T2D cases than controls. Independent of diabetic- and oral clinical status, dietary habits discriminated between individuals with and without oral impacts. The influence of meat and bread consumption on OIDP varied significantly according to T2D status.

## Background

Diabetes mellitus is a metabolic disorder characterised by chronic hyperglycaemia and disturbed carbohydrate, fat and protein metabolism and caused by defective insulin secretion, action, or both [1]. It is a major public health concern, with 380 million people afflicted worldwide. In 2014, The Middle East and North Africa had the highest age-adjusted global prevalence of diabetes (about 11%) and the prevalence in Sudan was about 18% [2]. Hyperglycaemia might lead to chronic, irreversible tissue damage. Long-term complications include nephropathy, retinopathy, neuropathy, cardiovascular diseases, peripheral vascular diseases, delayed healing and periodontal diseases [3].

Oral diseases such as periodontal diseases and tooth loss strongly influence food intake and limit the quality and quantity of food consumed [4]. Moreover, dietary habits are considered as modifiable risk factors that influence diabetes development and glycaemic control [5]. It has been reported that having a healthy diet rich in fruits and vegetables reduces the risk of metabolic and cardiovascular diseases and their underlying mechanisms such as oxidative stress, inflammation and insulin resistance [6, 7]. Another point to be considered is that modifying the dietary habits is a challenge in diabetes management, as reported by patients with diabetes [8].

In Sudan, several factors, such as changes in dietary habits and socio-economic status, have contributed to the increased prevalence of non-communicable diseases, including diabetes [9]. In many countries in The Middle East and North Africa, including Sudan, the traditional diet with high fibre and low fat and cholesterol content has changed towards a more westernised diet with high content of fat, sugar, and sodium [9]. It is reported that the daily per capita supplies of fat has increased in Sudan by 13%, from 1971 to 1997 [9].

It is evident that social and behavioural factors -including dietary habits- influence on individuals’ general health and quality of life [10]. Improving quality of life of type 2 diabetes (T2D) patients is regarded as an important target in diabetes management along with the medical care of this disease [11]. Health-related quality of life can be assessed by generic or disease specific questionnaires [12]. The multidimensional construct of oral health-related quality of life (OHRQoL) refers to the extent to which oral diseases impact on individual’s normal functioning, and addressing the psycho-social consequences of oral diseases [13]. Information about the influence of dietary habits on OHRQoL is limited; especially among patients with chronic diseases such as T2D. This study aimed to examine the influence of dietary habits on OHRQoL in individuals with and without T2D. It was hypothesised that dietary habits associate with OHRQoL independent of socio-demographics and clinical oral health indicators, and that this association differs between individuals with and without T2D.

## Methods

### Study area and participants

The present study was designed as a gender and age matched case-control study with a ratio of 2 controls per 1 case. Sample size was calculated to be 450 using Openepi version 3.01 with a power of 90%, two-sided confidence level of 95%, alpha level of 0.05, ratio of controls to cases of 2, percentage of exposed controls of 50% and an odds ratio (OR) of 2 as a minimum difference between groups to be detected. The study participants were enrolled between July and December 2012. In total, 447 participants were included in the present study (149 T2D cases and 298 controls without the disease). The T2D patients were recruited from the dental clinic at Jaber Abol’ez Diabetes Centre; the main public specialised referral hospital for patients with diabetes in Khartoum state, with a daily average of 250 patients visiting the centre [14]. Diabetes was diagnosed by specialist physicians at the centre according to the criteria of the American Diabetes Association [15]. The eligibility criteria for enrolment were: (i) being diagnosed with T2D more than one year ago (for T2D cases), (ii) having at least 10 remaining natural teeth, (iii) no medication with antibiotics or steroidal and/or non-steroidal anti-inflammatory agents over the past 3 weeks, (iv) no immunosuppressive chemotherapy, no current acute illness, no professional periodontal treatment during the past 6 months and no pregnancy or lactation.

Participants in the control group (subjects without T2D) were recruited from the outpatient dental clinic at the Khartoum Dental Teaching Hospital; the main public referral hospital providing dental services in Khartoum state. The same eligibility criteria as mentioned above for patients with T2D were applied for recruitment of controls, except for being diagnosed with diabetes. Subjects without diabetes were asked about signs and symptoms of diabetes and if suspected, they were referred for confirmation.

### Questionnaire-guided interview

All participants were interviewed in the dental clinic before the clinical examination by trained research assistants (three in total) using a structured questionnaire. The questionnaire consisted of three parts:

#### Socio-demographic characteristics

The first part of the questionnaire covered the socio-demographic characteristics such as: age, gender, marital status (married/unmarried), education (illiterate/literate), employment status (employed/unemployed) and ethnicity (Afro-Arab/African).

#### Food-frequency questionnaire

The second part of the questionnaire included questions about the dietary habits of the study participants. The questionnaire was adopted and modified from a study conducted in Sudan by Abdalla et al., [16]. Participants were asked about the frequency of their consumption of the following food items: milk, meat (white and red), eggs, vegetables, fruits, sweets and bread (reported as times/week). The variables were further dichotomised into high/low consumption with median values as a cut-off point: consumption of milk (high: >7 times/week, low: ≤7 times/week), consumption of meat (high: >8 times/week, low: ≤8 times/week), consumption of eggs (high: >2 times/week, low: ≤2 times/week), consumption of vegetables (high: >7 times/week, low: ≤7 times/week), consumption of fruits (high: >2 times/week, low: ≤2 times/week), consumption of sweets (high: >0 times/week, low: =0 times/week) and consumption of bread (high: >21 times/week, low: ≤21 times/week).

#### Oral impact on daily performance

The third part of the questionnaire gathered data about oral health-related quality of life using the Arabic version of the eight-item Oral Impact on Daily Performance inventory (OIDP) [17]. It includes the following items: “*During the past 6 months, how often have problems with your mouth and teeth caused you any difficulty with: eating and chewing food; speaking and pronouncing clearly; cleaning teeth; sleeping and relaxing; smiling and showing teeth without embarrassment; maintaining usual emotional state; carrying out major work and social role; and enjoying contact with people”*. Each item was assessed using a 5-point scale: never affected =0, less than once a month =1, once or twice a month =2, once or twice a week =3 and every, or nearly every day =4. Each OIDP frequency item was further dichotomised, yielding the categories: 0 = never affected (including the original category 0), 1 = affected (including the original categories 1–4). An additive sum score of the dichotomised frequency items was constructed (range 0–8) and dichotomised into OIDP = 0 (no daily performance affected) and OIDP >0 (at least one daily performance affected).

### Oral clinical examination

After completion of the interview, all participants underwent clinical examination of all teeth, except 3rd molars. A single examiner (HGM) conducted all examinations using standard dental chair, (N22) Color Coded Probe 2–4–6-8-10-12 mm markings, (NAB2) Color Coded Nabors Furcation Probe 3–6–9-12 mm markings, curette, mirror, probe, tweezers and cotton rolls. The examination comprised assessment of dental plaque using the Silness and Loe Index [18] (dichotomised into high/low using the median as a cut-off point), bleeding on probing (reported as percentage of teeth with bleeding on probing and dichotomised into high/low using the median as a cut-off point), tooth mobility index [19] (dichotomised into yes/no), periodontal probing depth (measured from the gingival margin to the base of the periodontal sulcus/pocket (mm) at four sites of each tooth (mesial, distal, buccal and lingual)) and dichotomised into (probing depth ≤ 4 mm, probing depth > 4 mm), root caries (yes/no) and decayed, missing and filled teeth index (DMFT) [20], dichotomised into (DMFT =0 and DMFT >0).

### Reliability test

The intra-examiner reliability of the solo examiner HGM was assessed by Cohen’s kappa (κ) to estimate coefficients of agreement of dichotomous judgments in two different sessions [21]. For this purpose, the oral examination was repeated for 20 randomly selected participants, within 2 weeks from the first session [22], and (κ) was calculated for tooth mobility (κ = 0.74), root caries (κ = 0.80), periodontal probing depth (κ = 0.88) and dental caries (κ = 1.00).

### Statistical analyses

Differences in the socio-demographic and clinical characteristics between the T2D cases and controls were assessed using independent sample-T test for normally distributed continues variables and chi-square test for categorical variables. Chi-square test was also used to test the difference in the prevalence of each of the OIDP items between T2D cases and controls, as well as to test the differences in consumption of food items between participants with OIDP =0 and those with OIDP >0, and the analysis was stratified according to the T2D status. Conditional logistic regression analysis was conducted to calculate the crude and adjusted odds ratios (ORs) and their 95% confidence intervals (CI) for the association between consumption of different food items (high/low) and OIDP (OIDP =0, OIDP >0); adjusting for diabetic status, dental plaque index, bleeding on probing, tooth mobility, root caries, periodontal probing depth and missing teeth. The statistical interaction between the consumption of each of the food items and T2D was tested. Finally, multiple variables logistic regression analysis was stratified according to the T2D status to examine the direction of interactions.

## Results

### Characteristics of the study participants according to diabetic status

The socio-demographic and clinical characteristics of the study participants are presented in Table 1. The mean age of both T2D cases and controls was 52.3 ± 10.4 years (range between 24 and 70 years) and 60% were women. There were no significant differences between T2D cases and controls regarding the socio-demographic characteristics investigated (marital status, education, employment status and ethnicity). Consumption of food items by diabetic status revealed that a lower proportion of T2D cases than of controls reported high consumption of milk, meat and sweets (*P* < 0.01), whereas a higher proportion of T2D cases than of controls reported high consumption of eggs and fruits (*P* < 0.05). The prevalences of dental plaque index, bleeding on probing, tooth mobility, periodontal probing depth > 4 mm, missing teeth and root caries were higher in patients with T2D than those without the disease (*P* < 0.01) (Table 1).

**Table 1:** Socio-demographic and clinical characteristics of the study participants

Variable	T2D cases (*n* = 149)	Controls (*n* = 298)	*P* value
Age, mean (SD)^a^	52.30 (10.41)	52.30 (10.40)	1.00
Gender, % (n)^b^			
Men	39.6 (59)	39.3 (117)	0.95
Women	60.4 (90)	60.7 (181)	
Marital status, % (n)^b^			
Married	92.0 (137)	95.0 (282)	0.21
Unmarried	8.0 (12)	5.0 (15)	
Education, % (n)^b^			
Illiterate	26.2 (39)	30.5 (91)	0.34
Literate	73.8 (110)	69.5 (207)	
Employment, % (n)^b^			
Employed	35.6 (53)	36.9 (110)	0.78
Unemployed	64.4 (96)	63.1 (188)	
Ethnic group, % (n)^b^			
Afro-Arab	75.3 (110)	78.6 (232)	0.43
African	24.7 (36)	21.4 (63)	
Consumption of milk, % (n)^b^			
High	67.1 (100)	83.2 (248)	<0.01
Low	32.9 (49)	16.8 (50)	
Consumption of meat, % (n)^b^			
High	40.3 (60)	60.7 (181)	<0.01
Low	59.7 (89)	39.3 (117)	
Consumption of eggs, % (n)^b^			
High	72.5 (108)	61.4 (183)	0.02
Low	27.5 (41)	38.6 (115)	
Consumption of vegetables, % (n)^b^			
High	85.2 (127)	86.6 (258)	0.70
Low	14.8 (22)	13.4 (40)	
Consumption of fruits, % (n)^b^			
High	36.9 (55)	22.8 (68)	<0.01
Low	63.1 (94)	77.2 (230)	
Consumption of sweets, % (n)^b^			
High	23.5 (35)	49.3 (147)	<0.01
Low	76.5 (114)	50.7 (151)	
Consumption of bread, % (n)^b^			
High	78.4 (116)	79.9 (236)	0.84
Low	21.6 (32)	20.8 (62)	
Dental plaque index, % (n)^b^			
High	75.2 (112)	36.9 (110)	<0.01
Low	24.8 (37)	63.1 (188)	
Bleeding on probing, % (n)^b^			
High	89.3 (133)	31.9 (95)	<0.01
Low	10.7 (16)	68.1 (203)	
Tooth mobility, % (n)^b^			
Yes	91.3 (136)	53.0 (158)	<0.01
No	8.7 (13)	47.0 (140)	
Periodontal probing depth, % (n)^b^			
> 4 mm	45.0 (67)	14.8 (44)	<0.01
≤ 4 mm	55.0 (82)	85.2 (254)	
Missing teeth, % (n)^b^			
≤ 21 teeth	25.5 (38)	9.4 (28)	<0.01
> 21 teeth	74.5 (111)	90.6 (270)	
DMFT, % (n)^b^			
Yes	94.6 (141)	95.6 (285)	0.81
No	5.4 (8)	4.4 (13)	
Root caries, % (n)^b^			
Yes	52.3 (78)	39.3 (117)	<0.01
No	47.7 (72)	60.7 (182)	
OIDP, % (n)^b^			
OIDP =0	64.4 (96)	86.2 (257)	<0.01
OIDP >0	35.6 (53)	13.8 (41)	

^a^Independent sample-T test

^b^Chi-square test

**Fig. 1:**
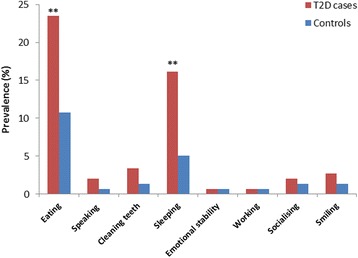
Prevalence of the eight OIDP items according to diabetic status. **P < 0.01 using chi-square test

Reporting any oral impact (OIDP > 0) was significantly more prevalent in T2D cases (35.6%) than their counterparts without the disease (13.8%), (*P* < 0.01) (Table 1). Among the eight OIDP items investigated in the present study, difficulty with eating and sleeping were more frequently reported by T2D cases (23.5% and 16.1%, respectively) than by the controls (10.7% and 5.0%, respectively) (*P* < 0.01) (Fig. 1).

### Dietary habits, OIDP and type 2 diabetes

The bivariate associations between the consumption of the different food items and OIDP are presented in Table 2, separately for T2D cases and controls. In T2D cases, there were no significant differences in the consumption of the food items between those with and without oral impacts. In the control group, low consumption of meat and vegetables was more frequently reported in participants with, than without oral impacts, whereas high consumption of sweets was most frequently reported in participants with oral impacts (*P* < 0.01).

**Table 2:** Consumption of the food items (high/low) according to OIDP status (OIDP = 0/OIDP >0), stratified according to T2D status

Consumption of food items, % (n)	Type 2 diabetes		Controls	
	OIDP = 0	OIDP >0	OIDP = 0	OIDP >0
Consumption of milk				
High	64.6 (62)	71.7 (38)	82.9 (213)	85.4 (35)
Low	35.4 (34)	28.3 (15)	17.1 (44)	14.6 (6)
Consumption of meat				
High	39.6 (38)	41.5 (22)	65.4 (168)	31.7 (13)**
Low	60.4 (58)	58.5 (31)	34.6 (89)	68.3 (28)
Consumption of eggs				
High	76.0 (73)	66.0 (35)	61.9 (159)	58.5 (24)
Low	24.0 (23)	34.0 (18)	38.1 (98)	41.5 (17)
Consumption of vegetables				
High	86.5 (83)	83.0 (44)	89.1 (229)	70.7 (29)**
Low	13.5 (13)	17.0 (9)	10.9 (28)	29.3 (12)
Consumption of fruits				
High	35.4 (34)	39.6 (21)	22.6 (58)	24.4 (10)
Low	64.6 (62)	60.4 (32)	77.4 (199)	75.6 (31)
Consumption of sweets				
High	20.8 (20)	28.3 (15)	45.5 (117)	73.2 (30)**
Low	79.2 (76)	71.7 (38)	54.5 (140)	26.8 (11)
Consumption of bread				
High	81.1 (77)	73.6 (39)	77.4 (199)	90.2 (37)
Low	18.9 (18)	26.4 (14)	22.6 (58)	9.8 (4)

**P < 0.01 using chi-square test

The results from crude and adjusted conditional regression analysis, regressing OIDP on consumption of food items are presented in Table 3. After adjusting for T2D status and the possible confounding variables, those with high consumption of milk and sweets, were more likely than those with low consumption to report any oral impact. The corresponding ORs were 1.23 (1.01–4.89) and 2.10 (1.08–4.09), respectively. Moreover, participants with low consumption of meat and vegetables were more likely than their counterparts with high consumption to report any oral impact. The corresponding ORs were 0.46 (0.25–0.83) and 0.38 (0.17–0.87), respectively. There was a significant two-way interaction between the consumption of meat and T2D status as well as between consumption of bread and T2D status. The stratified analyses presented in Table 4 revealed that the difference in meat consumption was not statistically significant in T2D cases (OR: 0.97, 95% CI: 0.47–2.00), while in the control group, participants with high consumption of meat were less likely to report any oral impact than their counterparts having low consumption (OR: 0.22, 95% CI: 0.10–0.46). The results of bread consumption were not statistically significant in either T2D cases or controls. The corresponding ORs were 0.62 (0.27–1.45) and 2.68 (0.91–7.89), respectively.

**Table 3:** OIDP regressed on consumption of food items, adjusted for T2D status and other possible confounding variables

Consumption of food items	Crude OR (95% CI)	*P* value	Adjusted OR (95% CI)^a^	*P* value
Consumption of milk				
High	1.03 (0.55–1.94)	0.11	1.23 (1.01–4.89)	0.04
Low	1		1	
Consumption of meat				
High	0.40 (0.22–0.70)	<0.01	0.46 (0.25–0.83)	0.01
Low	1		1	
Consumption of eggs				
High	0.98 (0.57–1.66)	0.93	0.80 (0.38–1.30)	0.25
Low	1		1	
Consumption of vegetables				
High	0.52 (0.26–1.04)	0.06	0.38 (0.17–0.87)	0.02
Low	1		1	
Consumption of fruits				
High	1.67 (0.91–3.05)	0.10	1.24 (0.62–2.48)	0.54
Low	1		1	
Consumption of sweets				
High	1.02 (0.61–1.73)	0.93	2.10 (1.08–4.09)	0.03
Low	1		1	
Consumption of bread				
High	1.14 (0.59–2.19)	0.70	1.55 (0.73–3.34)	0.25
Low	1		1	

^a^Conditional logistic regression analysis adjusting for: T2D status, dental plaque index, bleeding on probing, tooth mobility, root caries, periodontal probing depth and missing teeth

**Table 4:** OIDP regressed on consumption of food items stratified according to T2D status and adjusted for the possible confounding variables

Consumption of food items	Type 2 diabetes				Controls			
	Crude OR (95% CI)	*P* value	Adjusted OR (95% CI)*	*P* value	Crude OR (95% CI)	*P* value	Adjusted OR (95% CI)^a^	*P* value
Consumption of milk								
High	1.39 (0.67–2.88)	0.38	1.46 (0.68–3.14)	0.34	1.21 (0.48–3.04)	0.69	1.24 (0.49–3.18)	0.65
Low	1		1		1		1	
Consumption of meat								
High	1.08 (055–2.14)	0.82	0.97 (0.47–2.00)	0.94	0.25 (0.12–0.50)	<0.01	0.22 (0.10–0.46)	<0.01
Low	1		1		1		1	
Consumption of eggs								
High	0.61 (0.29–1.28)	0.19	0.57 (0.26–1.23)	0.15	0.87 (0.45–1.70)	0.68	0.89 (0.45–1.75)	0.73
Low	1		1		1		1	
Consumption of vegetables								
High	0.77 (0.30–1.93)	0.57	0.95 (0.35–2.55)	0.91	0.30 (0.14–0.64)	<0.01	0.31 (0.14–0.71)	<0.01
Low	1		1		1		1	
Consumption of fruits								
High	1.20 (0.60–2.39)	0.61	1.04 (0.49–2.21)	0.92	1.12 (0.51–2.40)	0.80	1.14 (0.53–2.49)	0.74
Low	1		1		1		1	
Consumption of sweets								
High	1.50 (0.69–3.25)	0.31	1.44 (0.64–3.22)	0.38	3.26 (1.57–6.79)	<0.01	3.42 (1.62–7.22)	<0.01
Low	1		1		1		1	
Consumption of bread								
High	0.65 (0.29–1.45)	0.29	0.62 (0.27–1.45)	0.28	2.70 (0.92–7.88)	0.07	2.68 (0.91–7.89)	0.07
Low	1		1		1		1	

^a^Logistic regression analysis adjusting for: dental plaque index, bleeding on probing, tooth mobility, root caries, periodontal probing depth and missing teeth

## Discussion

The present study shed, for the first time, light on the association between food consumption habits and OHRQoL in Sudanese adults with and without T2D; taking into consideration the potential variations in the socio-demographic- and clinical oral health indicators of the study population. The data indicated that independent of diabetic status; dietary habits significantly influenced the OIDP of the study participants. In addition, the impact of dietary habits on OIDP varied between those with and without T2D, having significant influence only among the control group.

Patients-centered factors are believed to be an important aspect in diabetes management [23]. These factors reflect the patients’ perception about their health condition regardless of the clinical measurements, which are often the main concern of health professionals. OIDP inventory is one of the tools by which OHRQoL can be assessed. The Arabic version of the OIDP utilised in the present study has been validated in the general population in Sudan, as well as among patients with mucocutaneous diseases [24, 25]. In general, the present study shows that T2D adversely influenced the OIDP, as a higher proportion of T2D cases than of the controls reported any oral impacts. A possible explanation for such finding is that they also lack knowledge and awareness about the oral complications of diabetes [26]. An important factor which might influence how patients with diabetes perceive oral health is that they are already burdened by other non-oral medical complications of the disease and are therefore, less likely to seek oral health care [27].

When the eight OIDP items were investigated, difficulty with eating and sleeping were more frequently reported by T2D cases than by the controls. These results are in contrast with a previous study from the UK, where diabetic status did not discriminate between patients with and without oral impacts as measured by the Oral Health Impact Profile-49 (OHIP-49) questionnaire [28]. Another study conducted in U. S adults with diabetes revealed that patients with diabetes were less likely to seek oral health care [29].Food-frequency questionnaire is a useful tool to assess how often each food item is consumed in a broad time frame such as times per day, week or month [30]. This tool has been used in case-control studies aimed to investigate the association between diet and chronic diseases [31–33]. The selection of food items included in the present study was based on the “traditional” meals most commonly consumed in Sudan; as the aim was not to assess the total dietary habits of the study participants. In general, the Sudanese food diet is essentially composed of cereals, meat, milk, eggs, fruits, and vegetables [34].

Fibre-rich diet including fruits and vegetables has great benefit to general health. It has been reported to be associated with decreased incidence and mortality of obesity-related diseases including cardiovascular diseases and diabetes [35]. A minimum of 5 servings of fruits and vegetables per day has been recommended by the World Health Organisation (WHO) as part of a person’s healthy diet [36]. However, the present study revealed that the weekly consumption of vegetables was below the median (7 times/week) in about 14.8% and 13.4% of the cases and controls, respectively; and only one participant in the control group reached the WHO recommended number of servings per day. Moreover, the weekly consumption of fruits was below the median (2 times/week) in about 63.1% and 77.2% of the cases and controls, respectively; and none of the study participants reached the WHO recommended portions. These figures indicate that the consumption of fruits and vegetables of the study participants is far less than the amount recommended by the WHO. This can be attributed to insufficient income, high prices and unavailability of these food items [34].

Consumption of sweets increases the blood glucose levels and is considered as one of the risk factors for developing diabetes [37, 38]. Therefore, patients with the disease are usually under strict dietary control with low sweets consumption. Nevertheless, about 23% of the T2D patients in the present study reported high consumption of sweets. Consistent with the present findings, a high consumption of sweets among T2D patients was reported in a study conducted in urban Ghana [39].

Most of studies that investigated the association between OHRQoL and food consumption have focused on chewing ability [40–44]. Khalifa et al., [43] assessed the association between perceived difficulty of chewing common Sudanese food items and OHRQoL among a sample of Sudanese adults ≥16 years old using the Oral Health Impact Profile (OHIP-14). They reported an OR of 2.33 (1.15–3.51) for those with chewing complain to report high OHIP scores. Another study from Japan concluded that patients’ perception of chewing ability is related to OHRQoL in complete denture wearers using the same tool (OHIP-14) [41]. OIDP has been utilised to assess the association between OHRQoL and eating habits in British population aged 65 years and older [44] and in older Tanzanians [42]. Both studies indicated that compromised chewing ability adversely influenced their daily performances. The present study is the first to investigate the influence of food consumption on OHRQoL in T2D patients using the OIDP inventory. It seems that dietary habits did not influence the OIDP prevalence reported by participants with T2D. In contrast, consumption of meat, vegetables and sweets significantly influence the OIDP prevalence reported by participants in the control group.

One of the strengths of this study is that it took into consideration the variations in the oral clinical indicators between the study groups when investigating the association between dietary habits and OIDP. The socioeconomic variation is an important factor when studying the dietary habits of a specific population. In the present study, both T2D cases and controls were recruited from dental clinics under the public health system, within the same geographic area (1 km apart). Therefore, we assume that both clinics have the same catchment area. Moreover, the level of education was included in the analysis as a proxy variable for measuring the socio-economic status of the study participants [45]. Food-frequency questionnaire might be subjected to misreporting and/or re-call bias [46]. Nevertheless, the study participants were interviewed to gather data about their dietary habits, thus reducing the influence of these sources of bias. Another point to be considered is that the present study is a hospital-based study, as both the cases and controls were attending dental clinics and they probably presented with more severe oral diseases and treatment needs compared to their non-dental attendee counterparts.

## Conclusions

Within the limitations of the present study, our data imply that oral impacts were more frequently reported by participants with T2D compared to those without diabetes. Independent of diabetic- and oral clinical status, dietary habits, in terms of consumption of meat, vegetables and sweets, discriminated between individuals with and without oral impacts. The influence of meat and bread consumption on OIDP varied significantly according to T2D status. The findings of the present study indicate the importance of maintaining balanced diet and the need for implementation of dietary counselling programmes for patients with T2D.

## Abbreviations

DMFT: Decayed missing and filled teeth index
OHRQoL: Oral health-related quality of life
OIDP: Oral impact on daily performance
T2D: Type 2 diabetes
κ: Cohen’s kappa

## References

[1] Alberti KG, Zimmet PZ (1998). Definition, diagnosis and classification of diabetes mellitus and its complications. Part 1: diagnosis and classification of diabetes mellitus provisional report of a WHO consultation. Diabet Med.

[2] International Diabetes Federation (2014). IDF Diabetes Atlas update poster.

[3] Sima C, Glogauer M (2013). Diabetes mellitus and periodontal diseases. Curr Diab Rep.

[4] Iwasaki M, Kimura Y, Yoshihara A, Ogawa H, Yamaga T, Takiguchi T (2015). Association between dental status and food diversity among older Japanese. Community Dent Health.

[5] Frank LK, Jannasch F, Kroger J, Bedu-Addo G, Mockenhaupt FP, Schulze MB (2015). A Dietary Pattern Derived by Reduced Rank Regression is Associated with Type 2 Diabetes in An Urban Ghanaian Population. Nutrients.

[6] Hu FB, Willett WC (2002). Optimal diets for prevention of coronary heart disease. JAMA.

[7] Folchetti LD, Monfort-Pires M, de Barros CR, Martini LA, Ferreira SR (2014). Association of fruits and vegetables consumption and related-vitamins with inflammatory and oxidative stress markers in prediabetic individuals. Diabetol Metab Syndr.

[8] Nagelkerk J, Reick K, Meengs L (2006). Perceived barriers and effective strategies to diabetes self-management. J Adv Nurs.

[9] Musaiger AO (2002). Diet and prevention of coronary heart disease in the Arab Middle East countries. Med Princ Pract.

[10] Rodriguez-Campello A, Jimenez-Conde J, Ois A, Cuadrado-Godia E, Giralt-Steinhauer E, Schroeder H (2014). Dietary habits in patients with ischemic stroke: a case-control study. PLoS One.

[11] Petterson T, Lee P, Hollis S, Young B, Newton P, Dornan T (1998). Well-being and treatment satisfaction in older people with diabetes. Diabetes Care.

[12] Rubin RR, Peyrot M (1999). Quality of life and diabetes. Diabetes Metab Res Rev.

[13] Sischo L, Broder HL (2011). Oral health-related quality of life: what, why, how, and future implications. J Dent Res.

[14] Makki Awouda FO, Elmukashfi TA, Hag Al-Tom SA (2014). Effects of health education of diabetic patient's knowledge at Diabetic Health Centers, Khartoum State, Sudan: 2007-2010. Glob J Health Sci.

[15] American Diabetes Association (2008). Diagnosis and classification of diabetes mellitus. Diabetes Care.

[16] Abdalla S, Leonhäuser I (2013). Dietary food consumption patterns in Sudan. J Agric Sci Rev.

[17] Adulyanon S, Sheiham A, Slade GD (1997). Oral Impact on Daily Performances. Measuring Oral Health and Quality of Life.

[18] Silness J, Loe H (1964). Periodontal disease in pregnancy. II. Correlation between oral hygiene and periodontal condition. Acta Odontol Scand.

[19] Miller SC. Textbook of Periodontia. 1 edn: Philadelphia: Blakiston; 1938.

[20] World Health Organization, 4 (1997). Oral Health Surveys, Basic Methods.

[21] Cohen J (1960). A coefficient of agreement for nominal scales. Educ Psychol Meas.

[22] Lang NP, Cullinan MP, Holborow DW, Heitz-Mayfield LJA, Giannobile WV, Burt BA, Genco RJ (2010). Examiner training: standardization and calibration in periodontal studies. Clinical research in oral health.

[23] Wandell PE (2005). Quality of life of patients with diabetes mellitus. An overview of research in primary health care in the Nordic countries. Scand J Prim Health Care.

[24] Nurelhuda NM, Ahmed MF, Trovik TA, Åstrøm AN (2010). Evaluation of oral health-related quality of life among Sudanese schoolchildren using Child-OIDP inventory. Health Qual Life Outcomes.

[25] Suliman NM, Johannessen AC, Ali RW, Salman H, Åstrøm AN (2012). Influence of oral mucosal lesions and oral symptoms on oral health related quality of life in dermatological patients: a cross sectional study in Sudan. BMC Oral Health.

[26] Masood Mirza K, Khan AA, Ali MM, Chaudhry S (2007). Oral health knowledge, attitude, and practices and sources of information for diabetic patients in Lahore, Pakistan. Diabetes Care.

[27] Allen EM, Ziada HM, O'Halloran D, Clerehugh V, Allen PF (2008). Attitudes, awareness and oral health-related quality of life in patients with diabetes. J Oral Rehabil.

[28] Irani FC, Wassall RR, Preshaw PM (2015). Impact of periodontal status on oral health-related quality of life in patients with and without type 2 diabetes. J Dent.

[29] Tomar SL, Lester A (2000). Dental and other health care visits among U.S. adults with diabetes. Diabetes Care.

[30] Cade J, Thompson R, Burley V, Warm D (2002). Development, validation and utilisation of food-frequency questionnaires - a review. Public Health Nutr.

[31] Slattery ML, Schumacher MC, Smith KR, West DW, Abd-Elghany N (1988). Physical activity, diet, and risk of colon cancer in Utah. Am J Epidemiol.

[32] Potischman N, Weiss HA, Swanson CA, Coates RJ, Gammon MD, Malone KE (1998). Diet during adolescence and risk of breast cancer among young women. J Natl Cancer Inst.

[33] Tzonou A, Lagiou P, Trichopoulou A, Tsoutsos V, Trichopoulos D (1998). Dietary iron and coronary heart disease risk: a study from Greece. Am J Epidemiol.

[34] Abdalla S, Taha A (2015). Analysis of food consumption patterns and diversity intake: using food frequency. Glob J Agric Food Secur.

[35] Boeing H, Bechthold A, Bub A, Ellinger S, Haller D, Kroke A (2012). Critical review: vegetables and fruit in the prevention of chronic diseases. Eur J Nutr.

[36] World Health Organization (2003). Diet, Nutrition and the Prevention of Chronic Diseases. Joint WHO/FAO Expert Consultation, WHO Technical Report Series no. 916.

[37] van Dam RM, Rimm EB, Willett WC, Stampfer MJ, Hu FB (2002). Dietary patterns and risk for type 2 diabetes mellitus in U.S. men. Ann Intern Med.

[38] Fung TT, Schulze M, Manson JE, Willett WC, Hu FB (2004). Dietary patterns, meat intake, and the risk of type 2 diabetes in women. Arch Intern Med.

[39] Frank LK, Kroger J, Schulze MB, Bedu-Addo G, Mockenhaupt FP, Danquah I (2014). Dietary patterns in urban Ghana and risk of type 2 diabetes. Br J Nutr.

[40] Lee IC, Yang YH, Ho PS, Lee IC (2014). Chewing ability, nutritional status and quality of life. J Oral Rehabil.

[41] Yamaga E, Sato Y, Minakuchi S (2013). A structural equation model relating oral condition, denture quality, chewing ability, satisfaction, and oral health-related quality of life in complete denture wearers. J Dent.

[42] Kida IA, Åstrøm AN, Strand GV, Masalu JR (2007). Chewing problems and dissatisfaction with chewing ability: a survey of older Tanzanians. Eur J Oral Sci.

[43] Khalifa N, Allen PF, Abu-bakr NH, Abdel-Rahman ME (2013). Chewing ability and associated factors in a Sudanese population. J Oral Sci.

[44] Sheiham A, Steele JG, Marcenes W, Tsakos G, Finch S, Walls AW (2001). Prevalence of impacts of dental and oral disorders and their effects on eating among older people; a national survey in Great Britain. Community Dent Oral Epidemiol.

[45] Adler NE, Rehkopf DH (2008). U.S. disparities in health: descriptions, causes, and mechanisms. Annu Rev Public Health.

[46] England CY, Andrews R, Jago R, Thompson JL (2014). Changes in reported food intake in adults with type 2 diabetes in response to a nonprescriptive dietary intervention. J Hum Nutr Diet.

